# Measuring advance care planning behavior in Dutch adults: translation, cultural adaptation and validation of the Advance Care Planning Engagement Survey

**DOI:** 10.1186/s12874-021-01389-5

**Published:** 2021-09-25

**Authors:** Doris van der Smissen, Agnes van der Heide, Rebecca L. Sudore, Judith A. C. Rietjens, Ida J. Korfage

**Affiliations:** 1 Department of Public Health, Erasmus MC, University Medical Center Rotterdam, P.O. Box 2040, 3000 Rotterdam, CA The Netherlands; 2 Division of Geriatrics, Department of Medicine, University of California, San Francisco School of Medicine, San Francisco, CA USA; 3 Geriatrics, Palliative, and Extended Care Service Line, San Francisco Veterans Affairs Medical, San Francisco, CA USA

**Keywords:** Advance care planning, Behavior change, Patient reported outcome measure, Translation, Dutch, Validation, Chronic disease

## Abstract

**Background:**

Advance care planning (ACP) enables people to define, discuss, and record preferences for treatment and care. Measures of ACP behavior are lacking in the Netherlands. We aimed to translate, culturally adapt and validate the 34-item ACP Engagement Survey into Dutch.

**Methods:**

Following validation guidelines, we tested content validity, internal consistency, reproducibility, construct validity, interpretability and criterion validity among persons with and without chronic disease.

**Results:**

Forward-backward translation indicated the need of only minor adaptations. Two hundred thirty-two persons completed baseline and retest surveys; 121 were aged ≥60 years. Persons with chronic disease (*n* = 151) considered the survey more valuable than those without (66 vs. 59, *p* < 0.001, scale of 20–100), indicating good content validity. Internal consistency (Cronbach’s alpha: 0.97) and reproducibility (intraclass correlation: 0.88) were good. Total ACP Engagement was higher among persons with chronic disease than those without (2.9 vs. 2.4, *p* < 0.01, scale of 1 to 5), indicating good psychometric support for construct validity and interpretability. Positive correlations of the ACP Engagement Survey and the General Self-Efficacy survey indicated good criterion validity (*p* < 0.05).

**Conclusions:**

This study provided good psychometric support for the validity and reliability of the Dutch 34-item ACP Engagement Survey. This instrument can be used to assess involvement in ACP in adults with and without chronic disease.

**Supplementary Information:**

The online version contains supplementary material available at 10.1186/s12874-021-01389-5.

## Background

Advance care planning (ACP) is a communication process which enables persons to define goals and preferences for future medical treatment and care and to discuss, record and review these preferences if appropriate [1]. Knowing patients’ preferences for treatment and care may support healthcare professionals in providing goal-concordant care [1]. People can engage in ACP at any moment in life, however, ACP can be more targeted when one’s health condition worsens [1]. The COVID-19 pandemic has made people become aware of their risk to suddenly become severely ill. Therefore, ACP is currently actively encouraged worldwide [2, 3].

To evaluate the effect of ACP, valid measurement instruments are required [1]. The Advance Care Planning (ACP) Engagement Survey, developed in the United States, is such an instrument. The survey, of which versions exist with 82, 55, 34, 15, 9 and 4 items, evaluates the effects of the entire ACP process [4, 5]. This is important because the focus of ACP has evolved from a sole focus of documenting preferences in an advance directive, to being an ongoing behavior change process in which individuals consider, discuss and record goals, values and preferences for treatment and care [1, 4]. The ACP Engagement Survey is grounded in social cognitive and behavior change theories to detect behavior change over a range of these behaviors [4, 5].

The survey has been shown to be reliable [4, 5], and to be sensitive to change in response to ACP interventions, such as the web-based programs ‘PREPARE’, ‘Making Your Wishes Known’, and ‘MyDirectives’ from the USA [6–10]. The survey will also be used to evaluate an ACP-General Practioner intervention in Belgium [11] and to evaluate home health care in Taiwan [12]. The survey may also be used to understand the public awareness of and engagement in ACP [4, 5]. The survey has been validated in English, Spanish, Japanese and Chinese [5, 13–15] and may also be useful and applicable in the Netherlands. Although the uptake of ACP has been relatively low in the Netherlands [16], Dutch policies increasingly encourage engagement in ACP, and an increasing number of Dutch ACP interventions have become available [1, 17]. However, instruments to measure ACP behavior are lacking in the Netherlands.

The 34-item version is the shortest version of the survey that still contains questions on all four domains and four subscales. The aim of this study is to translate, culturally adapt, and validate the 34-item version of the ACP Engagement Survey in Dutch.

## Methods

### The survey and translation

We aimed to translate, culturally adapt, and validate the 34-item version of the ACP Engagement Survey in Dutch. We applied international validation guidelines, namely the quality criteria of Terwee et al., during the validation process [18]. These criteria are developed to assess the quality of measurement properties of health status measures, such as the content validity, internal consistency, criterion validity and construct validity [18]. The 34-item version of the ACP Engagement Survey measures ACP behavior change considering four ACP domains: 1) surrogate decision makers; 2) values and quality of life, 3) flexibility in surrogate decision making, and 4) asking doctors questions [4]. ACP behavior change is measured with four subscales: knowledge about ACP (2 questions), contemplation about ACP (3 questions), self-efficacy for ACP (12 questions), and readiness for ACP (17 questions). Response options range from 1 = not at all to 5 = extremely for the knowledge and self-efficacy subscales and from 1 = never to 5 = a lot for the contemplation subscale. The response options to the readiness subscale range from 1 = I have never thought about it to 5 = I have already done it. The response option “I don’t know” is to be coded as missing. The total ACP Engagement score is the mean score of all responses in the survey.

### Translation from English into Dutch

We conducted forward-backward translation following the guidelines of Guillemin et al. [19]; three native Dutch speakers each independently translated the survey, reached consensus on a translation, a billingual speaker performed a backward-translation, and we decided on the final version with a committee of ACP researchers [19].

Three native Dutch speakers (DS, IK and AH) independently translated the items from English to Dutch, aiming to maintain the original meaning rather than literally translating the text. Discrepancies were resolved during consensus meetings with DS, IK and AH, and small adaptations were discussed with the developer of the ACP Engagement Survey (RS). After reaching consensus, the survey was translated backward by a bilingual speaker (native English and fluent in Dutch). After comparison of the backward translation to the original, final discrepancies were resolved. See Additional file 1 for an overview of the adaptations and Additional file 2 for the Dutch 34-item version.

### Validation

#### Content validity

We examined whether participants thought all important topics related to ACP were covered and whether topics were missing. To assess the content- and face validity of the ACP Engagement Survey we used the QQ-10 face validity survey [20], which has two domains. The Value domain (6 items) addresses whether respondents consider the survey relevant and easy to complete. The Burden domain (4 items) addresses whether the survey was too complicated, too long or upsetting. Response options have a 5-point scale, and the total score range is 20–100 [20]. We compared scores between respondents aged under 60 years versus 60 years and over, and between persons with versus without chronic disease.

Furthermore, we examined floor and ceiling effects for participants who filled in all 34 items of the ACP Engagement Survey without ever answering “I don’t know”. When more than 15% of these participants have the highest or lowest possible score, items are likely missing in the lower or upper end of the scales and content validity is considered limited [18].

#### Internal consistency

We assessed per subscale whether the items measured one underlying construct, by examining whether responses to the items were inter-correlated. We calculated Cronbach’s Alphas, which are considered sufficient when above 0.70, and preferably below 0.95 [18].

#### Reproducibility

We assessed test-retest reliability by asking participants to complete the survey twice; at baseline and after 1 week. We calculated the intraclass correlation coefficients (ICC_agreement_) using a two-way random effects model, resulting in a ratio ranging from 0 to 1 [18]. An intraclass correlation of 0.7 indicates good reliability [18].

To assess agreement between baseline and retest of the total ACP Engagement score and subscale scores, we conducted Bland Altman’s test [18]. Its first part consists of a one sample t-test with the difference scores (means retest minus means baseline) to examine whether the difference scores differed from 0; when non-significant (*p* > 0.05), agreement between the baseline and retest measurement is considered to be enhanced. The second part consists of a linear regression with the difference scores and the mean scores on the ACP Engagement Survey (baseline and retest); the absence of a significant difference is considered to indicate the absence of proportional bias. The third part consists of the calculation of the limits of agreement; the majority of differences in scores (95%) are expected to be within these limits, calculated as ‘mean change in scores +/- 1.96 x standard deviation (SD) of the changes’, and we checked these in the Bland Altman’s plot [18].

#### Criterion validity

To determine criterion validity, assessment of correlations of the instrument with a “gold standard” instrument is required [18]. Since such a gold standard instrument to measure ACP behavior is lacking in the Netherlands, we used the GSE measure on self-efficacy to assess these correlations [21]. This well-known, validated survey assesses concepts similar to the ACP Engagement Survey, namely self-efficacy (10 items, scale 1–5) [21, 22].

#### Construct validity

Construct validity is considered adequate when 75% of hypotheses about scores are supported [18]. We formulated the following hypotheses:

1. We hypothesize that the contemplation, readiness, and especially the self-efficacy subscales of the ACP Engagement Survey will be positively correlated with the GSE self-efficacy results [21].
2. Since ACP will probably be more relevant for persons with chronic disease, we expect them to have higher levels of ACP engagement, and especially for “Readiness”, than persons without chronic disease.
3. Since ACP will probably be more relevant for persons aged 60 years and over, we expect them to have higher levels of ACP engagement, and especially for “Readiness”, than persons aged below 60 years.
4. We expect the four subscales on knowledge, contemplation, self-efficacy and readiness to be inter-correlated.

### Study population and study design

For content, criterion and construct validation, we compared scores of persons with and without chronic diseases and people aged 60 and over and under age 60. Given the quality criteria about sample size (at least 50 persons per subgroup) [18] we estimated requiring 200 participants since we had 4 subgroups (persons with chronic disease below 60 years of age and over 60 years of age versus persons without chronic disease below 60 years of age and over 60 years of age).

### Data collection

Participants were recruited via a certified online Dutch research portal, called Flycatcher [23] and they provided written informed consent via this portal (double-active-opt-in) [23]. The online research portal is used for national representative research, and has over 10.000 Dutch members with various characteristics who voluntary signed up to participate in research [23]. Members can collect points for completing surveys that they can exchange for a gift card [23]. The portal is only accessible for the qualified moderators of the portal; the researchers had no access to personal data of the members/participants. We invited members with chronic disease and above the age of 18 years. We defined chronic disease as a disease that lasts at least 3 months, re-occurs regularly and is not (completely) curable, such as chronic obstructive pulmonary disease (COPD), Multiple Sclerosis (MS) and cancer. We did not include participants with a psychological disorder or dementia. We used purposive sampling by sending the questionnaire to comparable numbers of men and women, with diverse educational backgrounds, living in different areas of the Netherlands. Our participant sample was representative for Dutch persons of 18 years and over considering gender, educational level and residence [24].

The moderators of the Dutch research portal sent an email to a selection of members (with chronic disease, and above the age of 18 years) with a link to a questionnaire containing: 1) the 34-item version of the ACP Engagement Survey, 2) the QQ-10 face validity survey [20], and 3) the Dutch General Self-Efficacy scale (GSE) [21]. After 1 week, the participants were again asked to complete the ACP Engagement Survey and the GSE.

The local Medical Research Ethics Committee approved this study.

### Data analysis

For content validity, we conducted two independent t-tests to compare scores for the Value and Burden domains of the face validity survey (QQ-10) between respondents aged under 60 years versus 60 years and over, and between persons with versus without chronic disease. For criterion validity, we assessed “Spearman’s Rho” correlations of the subscales contemplation, self-efficacy and readiness with the GSE measure on self-efficacy [21]. For construct validity, we computed “Spearman’s Rho” correlation to test hypothesis 1 (correlation of the contemplation, self-efficacy and readiness subscales with the GSE self-efficacy survey) and hypothesis 4 (inter-correlation of the knowledge, contemplation, self-efficacy and readiness subscales). Furthermore, we conducted two one-way ANOVAs to test hypothesis 2 (differences between persons with versus without chronic disease on the ACP Engagement Survey; subscales and total ACP engagement score) and hypothesis 3 (differences between persons below 60 years of age versus 60 years and over and with versus without chronic disease on the ACP Engagement Survey; subscales and total ACP engagement score).

## Results

### Translation from English into Dutch

To culturally adapt the survey to the Dutch context, some adaptations were made. For instance, we changed “How confident are you…” into “Do you think you can…”; because the term “confident” was hard to translate into Dutch. We also decided to refer to “doctor” instead of “doctors”. In Dutch, there is little difference in meaning between “a little”, “somewhat” and “fairly” or between “once or twice”, “a few times” and “several times”. Therefore, we replaced the 5-point Likert scale by a 3-point scale for the subscales knowledge, contemplation and self-efficacy (coded as 1 = 1, 2 = 3, and 3 = 5). See Additional file 1 for all cultural adaptations.

### Participant characteristics

**Fig. 1:**
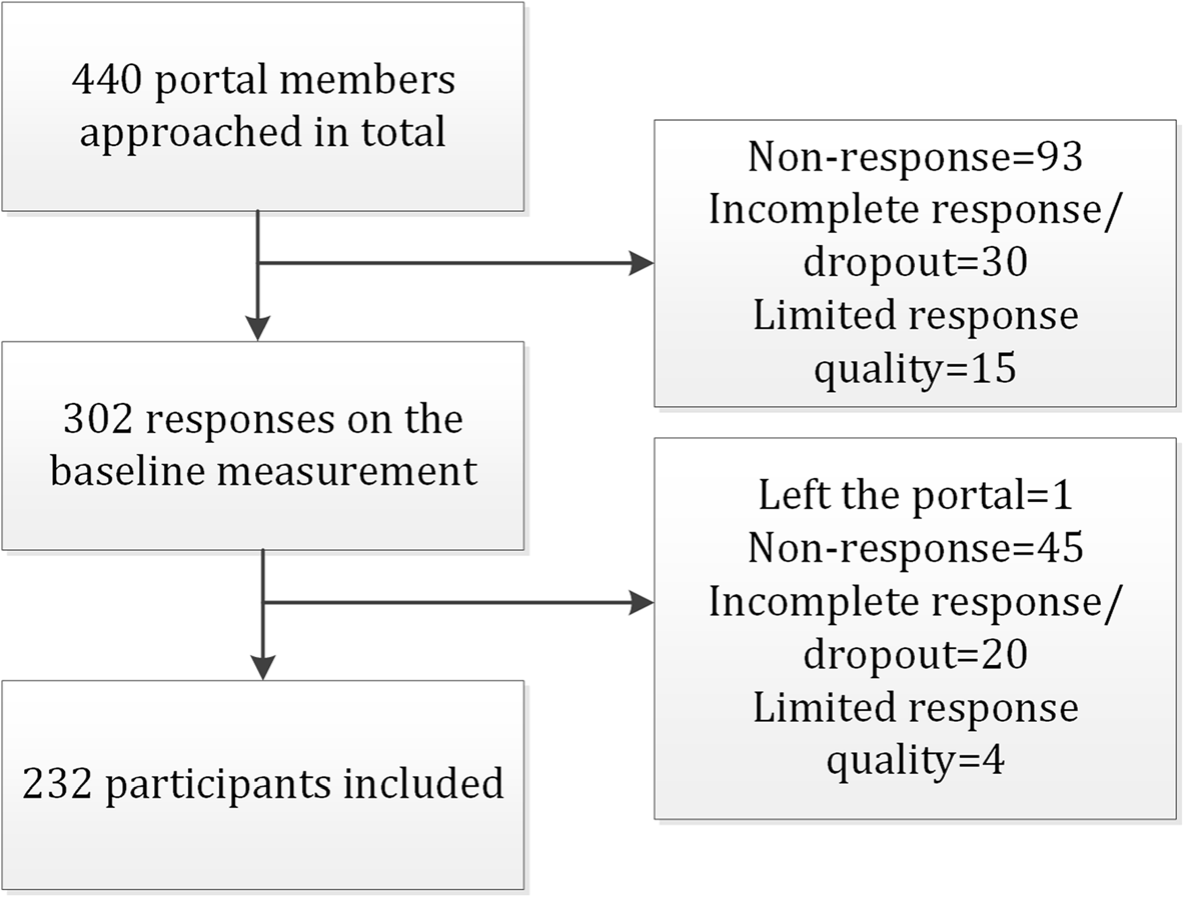
Flowchart of the inclusion of participants

The baseline measurement was completed by 302 of 440 approached participants (69%). The retest measurement was completed by 232 of 302 participants (77%), which were included in the data analyses (see Fig. 1).

Participants had a mean age of 58.9 years (age range: 18 to 93), 122 persons were male (53%). Sixty-four participants (28%) had low educational attainment (no education to lower general secondary education), 105 participants (45%) had medium educational attainment (secondary vocational education, higher general secondary education) and 63 participants (27%) had high educational attainment (higher vocational education or scientific education). Of all 232 participants, 111 were aged below 60 years (48%), 121 were aged 60 years and over (52%), and 123 had a chronic disease (53%). At baseline, it took participants on average 9.4 min (standard deviation = 9.8) to complete the ACP Engagement Survey, and at retest 11.8 min (standard deviation = 11.5).

### Content validity

Persons with chronic disease considered the ACP Engagement Survey more valuable than persons without chronic disease (score of 65.9 vs. 58.6, *p* < 0.001), no significant differences were found between persons aged below versus above 60 years (score of 61.2 vs. 63.6, *p* = 0.2), see Table 1. No differences were found for the burden domain.

**Table 1:** Results of the QQ-10 face validity survey (mean scores on a scale of 20–100)

	Mean score (SD^a^)	*P*-value
**Value domain, overall score**	62.5 (13.5)	
Age:		*0.2*
- Below 60 years	61.2 (12.9)	
- 60 years and over	63.6 (14.0)	
Chronic disease:		*0.001*
- With chronic disease	65.9 (14.0)	
- Without chronic disease	58.6 (11.8)	
**Burden domain (overall score)**	43.6 (13.0)	
Age:		*0.96*
- Below 60 years	43.6 (13.7)	
- 60 years and over	43.6 (12.4)	
Chronic disease:		*0.8*
- With chronic disease	43.4 (13.8)	
- Without chronic disease	43.8 (12.0)	

^a^*SD* Standard deviation

At baseline one of 232 participants (0.4%) had the lowest (i.e. 34 points), and 4 (1.7%) had the highest possible score (i.e. 170 points). At retest, no participants (0%) had the lowest, and 2 (0.9%) had the highest possible score. This indicates an absence of floor and ceiling effects, and thus enhances content validity.

### Internal consistency

At baseline and retest, Cronbach’s Alpha showed a high reliability for the total ACP Engagement score (0.97 and 0.97), and for the subscales of knowledge (0.80 and 0.75), contemplation (0.78 and 0.84), self-efficacy (0.94 and 0.94) and readiness (0.98 and 0.98).

### Reproducibility

The intraclass correlation coefficients showed good reliability for the total ACP Engagement score (0.88) and the subscales contemplation, self-efficacy, and readiness (0.82 to 0.87) and sub-optimal reliability for knowledge (0.64).

The Bland Altman’s t-tests showed no significant differences between test and retest (*p* > 0.05), except for the subscale contemplation at baseline. The linear regressions with the difference scores and the mean scores on the ACP Engagement Survey showed no significant differences either (*p* > 0.05). This indicates sufficient agreement between the baseline and retest measurement and the absence of proportional bias. The limits of agreement for the total ACP Engagement score and the Bland Altman’s plot, indicate a proportion of the differences in scores from baseline to retest (95%) were expected to be within this range; − 1.14 to 1.18. See Table 2 for all results related to reproducibility.

**Table 2:** Reproducibility of the test-retest measurements of the ACP Engagement Survey (34 items) and its subscales

	Intraclass correlation coefficient (95% CI^a^)	Mean change (SD^b^)	Limits of agreement^c^	Bland Altman’s: t-test; linear regression^d^
Total ACP Engagement (mean of all items)	0.88 (0.84–0.91)	0.019 (0.593)	−1.14 to 1.18	*p* = 0.6; *p* = 0.7
Subscale: Knowledge	0.64 (0.53–0.72)	−0.103 (1.318)	− 2.69 to 2.48	*p* = 0.2; *p* = 0.2
Subscale: Contemplation	0.84 (0.79–0.88)	−0.167 (0.923)	−1.98 to 1.64	*p* = 0.006; *p* = 0.7
Subscale: Self-efficacy	0.82 (0.76–0.86)	0.052 (0.724)	−1.37 to 1.47	*p* = 0.3; *p* = 0.6
Subscale: Readiness	0.87 (0.83–0.90)	−0.055 (0.725)	−1.48 to 1.37	*p* = 0.3; *p* = 0.5

^a^*CI* Confidence intervals

^b^*SD* Standard deviation

^c^Mean change in scores +/− 1.96 x standard deviation (SD) of the changes

^d^Linear regression with the difference score and the means

### Criterion validity

Providing psychometric support for criterion validity, we found that the subscales contemplation, self-efficacy and readiness correlated positively with the GSE self-efficacy survey at baseline (*p* < 0.05), see Table 3.

**Table 3:** Correlations of the subscales of the ACP Engagement Survey with the GSE self-efficacy survey

Subscale ACP Engagement Survey	Correlation coefficient (Spearman’s Rho)	
	Baseline measurement	*p*-value
Contemplation	*r* = 0.25	*p* < 0.001
Self-efficacy	*r* = 0.33	*p* < 0.001
Readiness	*r* = 0.16	*p* = 0.02

### Construct validity

In line with our first hypothesis, we found that the subscales contemplation, self-efficacy and readiness correlated positively with the GSE self-efficacy survey (*p* < 0.05). As expected, the strongest correlations were found for the subscale self-efficacy, see Table 3.

In line with the second hypothesis, the total ACP Engagement score at baseline was significantly higher for persons with chronic disease than for persons without chronic disease (3.0 vs. 2.4, *p* < 0.001). Furthermore, persons with chronic disease had higher scores than persons without chronic disease for the subscales contemplation (2.7 vs. 2.0, *p* < 0.001), self-efficacy (3.8 vs. 3.4, *p* = 0.003) and readiness (2.4 vs. 1.7, *p* < 0.001) but not for knowledge (*p* = 0.06).

The third hypothesis was partially supported, persons aged 60 years and over had higher scores than persons below 60 years for contemplation (2.8 vs. 2.0, *p* < 0.001) and readiness (2.3 vs. 1.9, *p* = 0.003). No significant differences were found between persons aged 60 years and over versus persons below 60 years for the total ACP Engagement score (*p* = 0.11), knowledge (*p* = 0.2) and self-efficacy (*p* = 0.053), see Table 4.

**Table 4:** Results of the ACP Engagement Survey at baseline by participant group

Participant group	With chronic disease	Without chronic disease	*P*-value	Age of 60 years and over	Age below 60 years	*P*-value
Total ACP Engagement (mean of the 34 items)	3.0 (1.0)	2.4 (0.7)	< 0.001	2.8 (0.9)	2.6 (0.8)	0.11
Subscale: Knowledge	3.2 (1.4)	2.8 (1.3)	0.06	3.1 (1.3)	2.9 (1.3)	0.2
Subscale: Contemplation	2.7 (1.3)	2.0 (1.1)	< 0.001	2.8 (1.3)	2.0 (1.1)	0.001
Subscale: Self-efficacy	3.8 (0.9)	3.4 (0.9)	0.003	3.5 (0.9)	3.8 (0.9)	0.053
Subscale: Readiness	2.4 (1.2)	1.7 (0.9)	< 0.001	2.3 (1.1)	1.9 (1.0)	0.003

In line with the fourth hypothesis, we found positive correlations between the four subscales (*p* < 0.001), see Table 5.

**Table 5:** Spearman’s correlation matrix of the ACP Engagement Survey subscales (baseline)

	Knowledge	Contemplation	Self-efficacy
Contemplation	0.53^a^		
Self-efficacy	0.29^a^	0.39^a^	
Readiness	0.45^a^	0.86^a^	0.36^a^

^a^*p* < 0.001

## Discussion

This study showed a sufficient content validity, internal validity, reliability and reproducibility of the Dutch 34-item version of the ACP Engagement Survey. Also, with 75% of hypotheses supported, a good construct validity was shown [18]. Significant correlations between the GSE self-efficacy survey and the subscales indicate sufficient criterion validity. Higher scores in ACP engagement for persons with chronic disease and persons aged 60 years and over provided psychometric support for the interpretability; we found higher scores for persons with versus without chronic disease regarding the total ACP Engagement score and the subscales contemplation, self-efficacy and readiness, with differences in scores ranging from 0.4 to 0.7 (mean difference score, scale 1 to 5). Persons aged 60 years and over versus below age 60 reported higher scores with a difference in scores of 0.8 for contemplation and 0.4 for readiness (mean difference score, scale 1 to 5), and no significant differences were found for the total ACP Engagement score, knowledge and self-efficacy.

In both our study and the US validation study [4], participants aged 60 years and over and persons with chronic disease had significantly higher levels of ACP engagement regarding several subscales, than participants with no chronic diseases and an age below 60 years. The US validation study shows that versions of the ACP Engagement Survey with 15, 9 and 4 items are also able to detect ACP behavior change [5]. These shorter versions consist of only self-efficacy and readiness items. The high Cronbach’s Alpha for the total ACP Engagement score (0.97), and the subscales of self-efficacy (0.94) and readiness (0.98), indicate that items could be deleted in the survey while maintaining its high internal consistency. Therefore, we expect shorter versions of the Dutch survey to also be able to detect ACP behavior change.

One limitation in our study was that since participants did not receive an intervention, we could not evaluate the responsiveness of the survey. Furthermore, the response format has been changed from a 5-point scale to a 3-point scale (coded as 1 = 1, 1 = 3 and 3 = 5) for the subscales knowledge, contemplation and self-efficacy and therefore differs from the original ACP Engagement Survey, which may complicate the comparison of findings across different studies. Since the participants were members of an online research portal, their level of computer skills/digital (health) literacy may be above the average of the Dutch population. A strength of this study is the application of the quality criteria of Terwee et al. [18]. We had a sufficient sample size, and participants had different ages, levels of education, and a variety of chronic diseases.

We recommend to study the effects of the Dutch ACP Engagement Survey in longitudinal research and to assess its responsiveness in, for example, randomized controlled trials to improve ACP engagement.

We developed the Dutch web-based ACP program ‘Explore your preferences for treatment and care’ (https://www.thuisarts.nl/keuzehulp/verken-uw-wensen-voor-zorg-en-behandeling), and we will use the 34-item ACP Engagement Survey to evaluate this program.

## Conclusions

Instruments to measure ACP behavior were lacking in the Netherlands. This study provided good psychometric support for the validity and reliability of the Dutch ACP Engagement Survey with 34 items. This instrument can be used to assess involvement in ACP in adults of all ages (ranging from 18 to 93 years old), with and without chronic disease. The survey can be used to evaluate the effect of ACP interventions, and it meets the need for a reliable Dutch measurement instrument for ACP.

## Supplementary Information

**Additional file 1.** Cultural adaptations to the Dutch 34-item version of the ACP Engagement Survey

**Additional file 2.** ACP Engagement vragenlijst 34 items

## Data Availability

The datasets generated and/or analysed during the current study are not publicly available because the data are confidential but are available from the corresponding author on reasonable request. The authors also translated other versions of the ACP Engagement survey with 82, 55, 15, 9 and 4 items from English to Dutch. These versions will be available through the following link: https://prepareforyourcare.org/resources.

## Abbreviations

ACP: Advance care planning
ACP Engagement Survey: Advance Care Planning Engagement Survey
CI: Confidence intervals
COPD: Chronic obstructive pulmonary disease
GSE: The Dutch General Self-Efficacy Scale
ICMJE: International Committee of Medical Journal Editors
MS: Multiple Sclerosis
SD: Standard deviation
US: United States
