# Supplementary material for: Measuring advance care planning behavior in Dutch adults: translation, cultural adaptation and validation of the Advance Care Planning Engagement Survey
Source: BMC Med Res Methodol. 2021 Sep 25;21:194. doi: 10.1186/s12874-021-01389-5 (PMC8467154; doi:10.1186/s12874-021-01389-5)
Supplement: Supplementary file 1 — Additional file 1. Cultural adaptations to the Dutch 34-item version of the ACP Engagement Survey [file 12874_2021_1389_MOESM1_ESM.pdf]

## Additional file 1

Cultural adaptations to the Dutch 34-item version of the ACP Engagement Survey.

| **Place of adaptation** | **Adaptation** |
|---|---|
| Entire survey | We changed “Doctors” into “Doctor”, because in the Netherlands people do not always have multiple doctors. |
| Self-efficacy items | The term ”confident” was hard to translate, we describe it as “Do you think you can…” instead of literally translating it. |
| Items with capitals, e.g.: “How ready are you to talk with your DOCTOR about who you want your medical decision maker to be?” | We wrote words with capitals in lower-case characters. In Dutch, it is uncommon to write survey questions in capitals. |
| Additional question to the Readiness items: “if yes, when did you do this?” | We added the verb, for instance: “if yes, when have you discussed this?” To clarify the question. |
| Questions in the “Values and quality of life” domain, e.g.:<br>“How confident are you that today you could talk with your medical decision maker about whether or not certain health situations would make your life not worth living?” | To also focus on what makes life worth living, we added “would” in addition to “would not”. |
| Introduction “Flexibility in decision making” domain. | Flexibility is a difficult term in Dutch. Therefore, we opted to describe it rather than using one word. |
| Names of subscale: KNOWLEDGE, THOUGHT ABOUT IT, SELF-EFFICACY and READINESS | We changed the name of the subscale into short sentences, e.g. “What you already know” for Knowledge, to enhance clarity in Dutch |
| Answer options of all subscales: answer option “refused” | The “Refused” option was deleted because participants can skip questions if they want. |
| Answer options of the subscales Knowledge, Contemplation and Self-efficacy | The 5-point Likert scales were reduced to 3 points: in Dutch, three answer options made more sense because of little differences in meaning. |
