# Supplementary material for: Measuring advance care planning behavior in Dutch adults: translation, cultural adaptation and validation of the Advance Care Planning Engagement Survey
Source: BMC Med Res Methodol. 2021 Sep 25;21:194. doi: 10.1186/s12874-021-01389-5 (PMC8467154; doi:10.1186/s12874-021-01389-5)
Supplement: Supplementary file 2 — Additional file 2. ACP Engagement vragenlijst 34 items [file 12874_2021_1389_MOESM2_ESM.docx]

## Additional file 2

| **ACP Engagement vragenlijst 34 items (*schuingedrukte vragen zijn optioneel****: de participant krijgt deze vragen alleen indien hij/zij “Ik heb het al gedaan” heeft ingevuld*)   \| We zullen u allereerst vragen stellen over uw ervaringen en opvattingen. De vragen kunnen gaan over dingen die u al heeft gedaan, of over dingen waar u nog nooit over heeft nagedacht. Beantwoord de vragen steeds zo eerlijk mogelijk.  *We zullen u vragen stellen over vier onderwerpen:*  1. Persoonlijk vertegenwoordigers  2. Nagaan wat er het meest toe doet in uw leven  3. Waarover uw persoonlijk vertegenwoordiger beslissingen mag nemen  4. Vragen stellen aan uw dokter \|  \| \| --- \| --- \| \| 1. Persoonlijk vertegenwoordigers  De volgende vragen gaan over persoonlijk vertegenwoordigers. Een persoonlijk vertegenwoordiger is een familielid of een vriend(in) die beslissingen voor u kan nemen als u daar zelf te ziek voor bent. Geef alstublieft zo eerlijk mogelijk antwoord, er zijn geen goede of foute antwoorden. \| Domein: Persoonlijk vertegenwoordigers \| \| 1. Weet u wat iemand een goede persoonlijk vertegenwoordiger maakt? \| Subschaal: Kennis \| \| - Ik weet dit niet - Ik weet dit een beetje - Ik weet dit zeker - Ik twijfel \| \| 2. Weet u wat voor medische beslissingen een persoonlijk vertegenwoordiger voor u zou kunnen nemen in de toekomst? \| Subschaal: Kennis \| \| - Ik weet dit niet - Ik weet dit een beetje - Ik weet dit zeker - Ik twijfel \| \| 3. Heeft u er wel eens over nagedacht wie uw persoonlijk vertegenwoordiger zou moeten zijn? \| Subschaal: Overweging \| \| - Ik heb hier nooit over nagedacht - Ik heb hier wel eens over nagedacht - Ik heb hier vaak over nagedacht - Ik weet het niet \| \| 4. Denkt u dat u iemand kunt vragen om uw persoonlijk vertegenwoordiger te worden? \| Subschaal: Eigen-effectiviteit \| \| - Ik denk dat ik dit niet kan - Ik denk dat ik dit kan - Ik denk dat ik dit heel goed kan - Ik weet het niet \| \| 5. Denkt u dat u met andere familie en vrienden kunt bespreken wie u als uw persoonlijk vertegenwoordiger wilt kiezen?  “Andere familie en vrienden” zijn mensen in uw leven die misschien ook een mening hebben over uw medische behandeling, maar die u niet als persoonlijk vertegenwoordiger zou kiezen. \| Subschaal: Eigen-effectiviteit \| \| - Ik denk dat ik dit niet kan - Ik denk dat ik dit kan - Ik denk dat ik dit heel goed kan - Ik weet het niet \| \| 6. Denkt u dat u met uw dokter kunt bespreken wie u als uw persoonlijk vertegenwoordiger wilt kiezen? \| Subschaal: Eigen-effectiviteit \| \| - Ik denk dat ik dit niet kan - Ik denk dat ik dit kan - Ik denk dat ik dit heel goed kan - Ik weet het niet \| \| 7. Bent u er klaar voor om iemand te vragen om uw persoonlijk vertegenwoordiger te worden? \| Subschaal: Bereidheid \| \| - Ik heb hier nooit over nagedacht - Ik heb hier wel over nagedacht, maar ik ben er nog niet aan toe - Ik denk dit binnen de komende 6 maanden te doen - Ik ga dit zeker in de komende maand doen - Ik heb het al gedaan - Ik weet het niet \| \| *Wanneer heeft u dat gedaan?* \| \| - *Minder dan 6 maanden geleden* - *Meer dan 6 maanden geleden* - *Ik weet het niet* \| \| 8. Bent u er klaar voor om met andere familie en vrienden te bespreken wie u als uw persoonlijk vertegenwoordiger wilt kiezen?  Nogmaals, “Andere familie en vrienden” zijn mensen in uw leven die misschien ook een mening hebben over uw medische behandeling, maar die u niet als persoonlijk vertegenwoordiger zou kiezen. \| Subschaal: Bereidheid \| \| - Ik heb hier nooit over nagedacht - Ik heb hier wel over nagedacht, maar ik ben er nog niet aan toe - Ik denk dit binnen de komende 6 maanden te doen - Ik ga dit zeker in de komende maand doen - Ik heb het al gedaan - Ik weet het niet \| \| *Wanneer heeft u dat gedaan?* \| \| - *Minder dan 6 maanden geleden* - *Meer dan 6 maanden geleden* - *Ik weet het niet* \| \| 9. Bent u er klaar voor om met uw dokter te bespreken wie u als uw persoonlijk vertegenwoordiger wilt kiezen? \| Subschaal: Bereidheid \| \| - Ik heb hier nooit over nagedacht - Ik heb hier wel over nagedacht, maar ik ben er nog niet aan toe - Ik denk er over om dit binnenkort een keer te doen - Ik ga dit zeker doen zodra ik mijn dokter zie - Ik heb het al gedaan - Ik weet het niet \| \| *Wanneer heeft u dat gedaan?* \| \| - *Minder dan 6 maanden geleden* - *Meer dan 6 maanden geleden* - *Ik weet het niet* \| \| 10. Bent u er klaar voor om schriftelijk vast te leggen wie u als uw persoonlijk vertegenwoordiger wilt kiezen? \| Subschaal: Bereidheid \| \| - Ik heb hier nooit over nagedacht - Ik heb hier wel over nagedacht, maar ik ben er nog niet aan toe - Ik denk dit binnen de komende 6 maanden te doen - Ik ga dit zeker in de komende maand doen - Ik heb het al gedaan - Ik weet het niet \|  \|  \| \| *Wanneer heeft u dat gedaan?* \| \| - *Minder dan 6 maanden geleden* - *Meer dan 6 maanden geleden* - *Ik weet het niet* \| \| 1. Wat er het meest toe doet in uw leven.   Nu willen we u een aantal vragen stellen over hoe u denkt over uw kwaliteit van leven. Bijvoorbeeld over hoe u wilt leven en hoe u niet wilt leven. Sommige mensen vinden dat het leven het in alle omstandigheden waard is om geleefd te worden, ook als ze bijvoorbeeld in coma zijn of niet meer uit bed kunnen komen. Andere mensen vinden dat er omstandigheden kunnen zijn waarin zij niet verder zouden willen leven. Beantwoord de vragen alstublieft weer zo eerlijk mogelijk. Er zijn geen goede of foute antwoorden. \| Domein: Wat er het meest toe doet in uw leven \| \| 11. Denkt u dat u met uw persoonlijk vertegenwoordiger kunt bespreken of er bepaalde gezondheidstoestanden zijn die voor u het leven wel of niet de moeite waard zouden maken? \| Subschaal: Eigen-effectiviteit \| \| - Ik denk dat ik dit niet kan - Ik denk dat ik dit kan - Ik denk dat ik dit heel goed kan - Ik weet het niet \| \| 12. Denkt u dat u met uw andere familie en vrienden kunt bespreken of er bepaalde gezondheidstoestanden zijn die voor u het leven wel of niet de moeite waard zouden maken? \| Subschaal: Eigen-effectiviteit \| \| - Ik denk dat ik dit niet kan - Ik denk dat ik dit kan - Ik denk dat ik dit heel goed kan - Ik weet het niet \| \| 13. Denkt u dat u met uw dokter kunt bespreken of er bepaalde gezondheidstoestanden zijn die voor u het leven wel of niet de moeite waard zouden maken? \| Subschaal: Eigen-effectiviteit \| \| - Ik denk dat ik dit niet kan - Ik denk dat ik dit kan - Ik denk dat ik dit heel goed kan - Ik weet het niet \| \| 14. Bent u er aan toe om bij uzelf na te gaan welke gezondheidstoestanden voor u het leven wel of niet de moeite waard zouden maken? \| Subschaal: Bereidheid \| \| - Ik heb hier nooit over nagedacht - Ik heb hier wel over nagedacht, maar ik ben er nog niet aan toe - Ik denk dit binnen de komende 6 maanden te doen - Ik ga dit zeker in de komende maand doen - Ik heb het al gedaan - Ik weet het niet \| \| *Wanneer heeft u dat gedaan?* \| \| - *Minder dan 6 maanden geleden* - *Meer dan 6 maanden geleden* - *Ik weet het niet* \| \| 15. Bent u er klaar voor om met uw persoonlijk vertegenwoordiger te bespreken welke gezondheidstoestanden voor u het leven wel of niet de moeite waard zouden maken? \| Subschaal: Bereidheid \| \| - Ik heb hier nooit over nagedacht - Ik heb hier wel over nagedacht, maar ik ben er nog niet aan toe - Ik denk dit binnen de komende 6 maanden te doen - Ik ga dit zeker in de komende maand doen - Ik heb het al gedaan - Ik weet het niet \| \| *Wanneer heeft u dat gedaan?* \| \| - *Minder dan 6 maanden geleden* - *Meer dan 6 maanden geleden* - *Ik weet het niet* \| \| 16. Bent u er klaar voor om met andere familie en vrienden te bespreken of er bepaalde gezondheidstoestanden zijn die voor u het leven wel of niet de moeite waard zouden maken? \| Subschaal: Bereidheid \| \| - Ik heb hier nooit over nagedacht - Ik heb hier wel over nagedacht, maar ik ben er nog niet aan toe - Ik denk dit binnen de komende 6 maanden te doen - Ik ga dit zeker in de komende maand doen - Ik heb het al gedaan - Ik weet het niet \| \| *Wanneer heeft u dat gedaan?* \| \| - *Minder dan 6 maanden geleden* - *Meer dan 6 maanden geleden* - *Ik weet het niet* \| \| 17. Bent u er klaar voor om met uw dokter te bespreken of er bepaalde gezondheidstoestanden zijn die voor u het leven wel of niet de moeite waard zouden maken? \| Subschaal: Bereidheid \| \| - Ik heb hier nooit over nagedacht - Ik heb hier wel over nagedacht, maar ik ben er nog niet aan toe - Ik denk er over om dit binnenkort een keer te doen - Ik ga dit zeker doen zodra ik mijn dokter zie - Ik heb het al gedaan - Ik weet het niet \| \| *Wanneer heeft u dat gedaan?* \| \| - *Minder dan 6 maanden geleden* - *Meer dan 6 maanden geleden* - *Ik weet het niet* \| \| We gaan nu over op een ander onderwerp. De vorige vragen gingen over gezondheidstoestanden die voor mensen het leven wel of niet de moeite waard zouden maken. De volgende vragen gaan over bepaalde medische behandelingen die mensen wel of niet zouden willen ondergaan als zij ernstig ziek of in de laatste levensfase zijn. Sommige mensen weten bijvoorbeeld dat zij wel kunstmatig beademd zouden willen worden. Andere mensen weten dat zij dat nooit zouden willen. Beantwoord de vragen over medische behandeling alstublieft zo eerlijk mogelijk. Er zijn geen goede of foute antwoorden. \|  \| \| 18. Heeft u er wel eens over nagedacht om met andere familie en vrienden te bespreken wat voor zorg u zou willen als u ernstig ziek bent of aan het einde van uw leven? \| Subschaal: Overweging \| \| - Ik heb hier nooit over nagedacht - Ik heb hier wel eens over nagedacht - Ik heb hier vaak over nagedacht - Ik weet het niet \| \| 19. Denkt u dat u met uw persoonlijk vertegenwoordiger kunt bespreken wat voor zorg u zou willen als u ernstig ziek bent of aan het einde van uw leven? \| Subschaal: Eigen-effectiviteit \| \| - Ik denk dat ik dit niet kan - Ik denk dat ik dit kan - Ik denk dat ik dit heel goed kan - Ik weet het niet \| \| 20. Denkt u dat u met andere familie en vrienden kunt bespreken wat voor zorg u zou willen als u ernstig ziek bent of aan het einde van uw leven? \| Subschaal: Eigen-effectiviteit \| \| - Ik denk dat ik dit niet kan - Ik denk dat ik dit kan - Ik denk dat ik dit heel goed kan - Ik weet het niet \| \| 21. Denkt u dat u met uw dokter kunt bespreken wat voor zorg u zou willen als u ernstig ziek bent of aan het einde van uw leven? \| Subschaal: Eigen-effectiviteit \| \| - Ik denk dat ik dit niet kan - Ik denk dat ik dit kan - Ik denk dat ik dit heel goed kan - Ik weet het niet \| \| 22. Bent u er klaar voor om te beslissen wat voor medische zorg u zou willen als u ernstig ziek bent of aan het einde van uw leven? \| Subschaal: Bereidheid \| \| - Ik heb hier nooit over nagedacht - Ik heb hier wel over nagedacht, maar ik ben er nog niet aan toe - Ik denk dit binnen de komende 6 maanden te doen - Ik ga dit zeker in de komende maand doen - Ik heb het al gedaan - Ik weet het niet \| \| *Wanneer heeft u dat gedaan?* \| \| - *Minder dan 6 maanden geleden* - *Meer dan 6 maanden geleden* - *Ik weet het niet* \| \| 23. Bent u er klaar voor om met uw persoonlijk vertegenwoordiger te bespreken wat voor medische zorg u zou willen als u ernstig ziek bent of aan het einde van uw leven? \| Subschaal: Bereidheid \| \| - Ik heb hier nooit over nagedacht - Ik heb hier wel over nagedacht, maar ik ben er nog niet aan toe - Ik denk dit binnen de komende 6 maanden te doen - Ik ga dit zeker in de komende maand doen - Ik heb het al gedaan - Ik weet het niet \| \| *Wanneer heeft u dat gedaan?* \|  \| \| - *Minder dan 6 maanden geleden* - *Meer dan 6 maanden geleden* - *Ik weet het niet* \| \| 24. Bent u er klaar voor om met andere familie en vrienden te bespreken wat voor medische zorg u zou willen als u ernstig ziek bent of aan het einde van uw leven? \| Subschaal: Bereidheid \| \| - Ik heb hier nooit over nagedacht - Ik heb hier wel over nagedacht, maar ik ben er nog niet aan toe - Ik denk dit binnen de komende 6 maanden te doen - Ik ga dit zeker in de komende maand doen - Ik heb het al gedaan - Ik weet het niet \| \| *Wanneer heeft u dat gedaan?* \| \| - *Minder dan 6 maanden geleden* - *Meer dan 6 maanden geleden* - *Ik weet het niet* \| \| 25. Bent u er klaar voor om met uw dokter te bespreken wat voor medische zorg u zou willen als u ernstig ziek bent of aan het einde van uw leven? \| Subschaal: Bereidheid \| \| - Ik heb hier nooit over nagedacht - Ik heb hier wel over nagedacht, maar ik ben er nog niet aan toe - Ik denk er over om dit binnenkort een keer te doen - Ik ga dit zeker doen zodra ik mijn dokter zie - Ik heb het al gedaan - Ik weet het niet \| \| *Wanneer heeft u dat gedaan?* \| \| - *Minder dan 6 maanden geleden* - *Meer dan 6 maanden geleden* - *Ik weet het niet* \| \| 26. Bent u er klaar voor om schriftelijk vast te leggen wat voor medische zorg u zou willen als u ernstig ziek bent of aan het einde van uw leven? \| Subschaal: Bereidheid \| \| - Ik heb hier nooit over nagedacht - Ik heb hier wel over nagedacht, maar ik ben er nog niet aan toe - Ik denk dit binnen de komende 6 maanden te doen - Ik ga dit zeker in de komende maand doen - Ik heb het al gedaan - Ik weet het niet \| \| *Wanneer heeft u dat gedaan?* \| \| - *Minder dan 6 maanden geleden* - *Meer dan 6 maanden geleden* - *Ik weet het niet* \| \| 1. Waarover uw persoonlijk vertegenwoordiger beslissingen mag nemen.   Nu vragen we u waarover uw persoonlijk vertegenwoordiger beslissingen mag nemen wanneer u dit zelf niet kunt. \| Domein: Waarover uw persoonlijk vertegenwoordiger beslissingen mag nemen. \| \| 27. Heeft u er wel eens over nagedacht om met uw persoonlijk vertegenwoordiger te bespreken waarover hij of zij beslissingen mag nemen? \| Subschaal: Overweging \| \| - Ik heb hier nooit over nagedacht - Ik heb hier wel eens over nagedacht - Ik heb hier vaak over nagedacht - Ik weet het niet \| \| 28. Denkt u dat u met andere familie en vrienden kunt bespreken waarover uw persoonlijk vertegenwoordiger beslissingen mag nemen? \| Subschaal: Eigen-effectiviteit \| \| - Ik denk dat ik dit niet kan - Ik denk dat ik dit kan - Ik denk dat ik dit heel goed kan - Ik weet het niet \| \| 29. Denkt u dat u met uw dokter kunt bespreken waarover uw persoonlijk vertegenwoordiger beslissingen mag nemen? \| Subschaal: Eigen-effectiviteit \| \| - Ik denk dat ik dit niet kan - Ik denk dat ik dit kan - Ik denk dat ik dit heel goed kan - Ik weet het niet \| \| 30. Bent u er klaar voor om met uw persoonlijk vertegenwoordiger te bespreken waarover hij of zij beslissingen mag nemen? \| Subschaal: Bereidheid \| \| - Ik heb hier nooit over nagedacht - Ik heb hier wel over nagedacht, maar ik ben er nog niet aan toe - Ik denk dit binnen de komende 6 maanden te doen - Ik ga dit zeker in de komende maand doen - Ik heb het al gedaan - Ik weet het niet \| \| *Wanneer heeft u dat gedaan?* \| \| - *Minder dan 6 maanden geleden* - *Meer dan 6 maanden geleden* - *Ik weet het niet* \| \| 31. Bent u er klaar voor om met uw dokter te bespreken waarover uw persoonlijk vertegenwoordiger beslissingen mag nemen? \| Subschaal: Bereidheid \| \| - Ik heb hier nooit over nagedacht - Ik heb hier wel over nagedacht, maar ik ben er nog niet aan toe - Ik denk er over om dit binnenkort een keer te doen - Ik ga dit zeker doen zodra ik mijn dokter zie - Ik heb het al gedaan - Ik weet het niet \| \| *Wanneer heeft u dat gedaan?* \| \| - *Minder dan 6 maanden geleden* - *Meer dan 6 maanden geleden* - *Ik weet het niet* \| \| 32. Bent u er klaar voor om schriftelijk vast te leggen waarover uw persoonlijk vertegenwoordiger beslissingen mag nemen? \| Subschaal: Bereidheid \| \| - Ik heb hier nooit over nagedacht - Ik heb hier wel over nagedacht, maar ik ben er nog niet aan toe - Ik denk dit binnen de komende 6 maanden te doen - Ik ga dit zeker in de komende maand doen - Ik heb het al gedaan - Ik weet het niet \| \| *Wanneer heeft u dat gedaan?* \| \| - *Minder dan 6 maanden geleden* - *Meer dan 6 maanden geleden* - *Ik weet het niet* \| \| 1. Vragen stellen aan uw dokter. \| Domein: Vragen stellen aan uw dokter \| \| 33. Denkt u dat u de goede vragen aan uw dokter kunt stellen om een goede medische beslissing te kunnen nemen? \| Subschaal: Eigen-effectiviteit \| \| - Ik denk dat ik dit niet kan - Ik denk dat ik dit kan - Ik denk dat ik dit heel goed kan - Ik weet het niet \| \| 34. Bent u er klaar voor om vragen aan uw dokter te stellen om een goede medische beslissing te kunnen nemen? \| Subschaal: Bereidheid \| \| - Ik heb hier nooit over nagedacht - Ik heb hier wel over nagedacht, maar ik ben er nog niet aan toe - Ik denk dit binnen de komende 6 maanden te doen - Ik ga dit zeker in de komende maand doen - Ik heb het al gedaan - Ik weet het niet \| \| *Wanneer heeft u dat gedaan?* \| \| - *Minder dan 6 maanden geleden* - *Meer dan 6 maanden geleden* - *Ik weet het niet* \| |
| --- | --- | --- | --- | --- | --- | --- | --- | --- | --- | --- | --- | --- | --- | --- | --- | --- | --- | --- | --- | --- | --- | --- | --- | --- | --- | --- | --- | --- | --- | --- | --- | --- | --- | --- | --- | --- | --- | --- | --- | --- | --- | --- | --- | --- | --- | --- | --- | --- | --- | --- | --- | --- | --- | --- | --- | --- | --- | --- | --- | --- | --- | --- | --- | --- | --- | --- | --- | --- | --- | --- | --- | --- | --- | --- | --- | --- | --- | --- | --- | --- | --- | --- | --- | --- | --- | --- | --- | --- | --- | --- | --- | --- | --- | --- | --- | --- | --- | --- | --- | --- | --- | --- | --- | --- | --- | --- | --- | --- | --- | --- | --- | --- | --- | --- | --- | --- | --- | --- | --- | --- | --- | --- | --- | --- | --- | --- | --- | --- | --- | --- | --- | --- | --- | --- | --- | --- | --- | --- | --- | --- | --- | --- | --- | --- | --- | --- | --- | --- | --- | --- | --- |
